# Network Pharmacology Perspectives on Cerebral Palsy: Mechanistic Insights From Ayurvedic Formulations With Emphasis on Kalyanaka Ghritam

**DOI:** 10.7759/cureus.107376

**Published:** 2026-04-20

**Authors:** Preetham Pai, Parag Bajirao Gaikwad, Sreelatha Shetty, Roopesh Shridhar Jadhav

**Affiliations:** 1 Department of Kaumarabhritya-Balroga, Bharati Vidyapeeth Deemed University, Pune, IND; 2 Department of Pediatrics, Maharashtra University of Health Sciences (MUHS), Nashik, IND; 3 Department of Ayurveda, Tilak Maharashtra Vidyapeeth, Pune, IND; 4 Department of Dravyaguna Vigyan, Pharate Patil Ayurvedic Medical College Mandavgan Pharata, Pune, IND

**Keywords:** ayurveda, cerebral palsy, kalyanaka ghritam, network pharmacology, neurodevelopment

## Abstract

Cerebral palsy is a non-progressive neurodevelopmental disorder arising from early brain injury and characterised by persistent motor dysfunction accompanied by cognitive, sensory, and behavioural impairments. Current management strategies remain largely supportive and symptomatic, reflecting the limited availability of interventions capable of addressing underlying molecular and network-level pathology. Increasing recognition of cerebral palsy as a multifactorial condition involving neuroinflammation, oxidative stress, synaptic dysregulation, and impaired neurodevelopment underscores the need for multi-target therapeutic frameworks. This narrative review adopts a structured methodology involving systematic database searches (PubMed, Scopus, Web of Science, Google Scholar, and AYUSH Research Portal) covering literature from 2015 to 2025, with predefined inclusion criteria focusing on phytochemical profiling, molecular targets, signalling pathways, and translational relevance to neurodevelopmental disorders. Particular emphasis was placed on studies integrating network pharmacology approaches to map compound-target-pathway interactions relevant to cerebral palsy. This narrative review aims to examine network pharmacology perspectives on Ayurvedic formulations, with emphasis on Kalyanaka Ghritam, to elucidate mechanistic relevance in cerebral palsy. A narrative synthesis of literature published between 2015 and 2025 was conducted using biomedical and traditional medicine databases, focusing on phytochemical constituents, molecular targets, biological pathways, and translational evidence. Phytochemical constituents relevant to this disease context include flavonoids (e.g., quercetin, kaempferol, luteolin) with antioxidant and anti-inflammatory properties; alkaloids and glycosides involved in neuromodulation of cholinergic, glutamatergic, and GABAergic systems; terpenoids contributing to mitochondrial protection and membrane stabilisation; and phenolic compounds and saponins that enhance neurotrophic signalling and mitigate apoptotic pathways. These bioactive classes collectively target key pathological processes in cerebral palsy, including oxidative stress, neuroinflammation, excitotoxicity, and impaired synaptic plasticity. Integrated analysis highlights that Kalyanaka Ghritam contains diverse bioactive compounds capable of modulating interconnected gene and protein networks associated with inflammatory regulation, redox homeostasis, neurotrophic signalling, synaptic plasticity, and neuroprotection. Such systems-level modulation aligns with the complex pathobiology of cerebral palsy and supports its potential adjunctive role within integrative care models. Network pharmacology emerges as a robust methodological bridge linking traditional therapeutic knowledge with contemporary systems biology, facilitating rational evaluation of multi-component interventions. Further experimental validation, standardisation, and controlled clinical studies remain essential to advance translational application and clinical integration.

## Introduction and background

Cerebral palsy is a heterogeneous set of irreversible neurodevelopmental disorders, which are defined by the limitation of movement, posture, and motor coordination, often with disruptions of sensation, thinking, communication, and behaviour [1]. The disease is caused by non-progressive damage of the developing brain, which is usually associated with hypoxia-ischemia in the foetus, perinatal inflammation, preterm birth, and early metabolic insults [2]. Recent global burden estimates indicate that cerebral palsy remains one of the most common causes of childhood disability worldwide, with prevalence ranging between two and three per 1000 live births globally, and higher rates reported in low- and middle-income countries due to disparities in perinatal care, neonatal survival, and access to rehabilitation services. Recent analyses also highlight an increasing absolute number of affected individuals attributable to improved survival of preterm and high-risk neonates, thereby amplifying long-term healthcare, social, and economic burden. Additionally, recent global health datasets further emphasise that cerebral palsy contributes substantially to years lived with disability (YLDs) in paediatric populations, reflecting its persistent functional impact across the lifespan and reinforcing the need for disease-modifying and systems-level therapeutic strategies.

The prevalence at a global level is maintaining a steady number around two to three per 1000 live births, which has been causing a perpetual burden on the individuals and their families as well as the healthcare systems [3]. The present clinical treatment is centred on symptomatic treatment elements, including physiotherapy, pharmacological management of spasticity, orthopaedic surgical interventions, and assistive neurorehabilitation, whereas disease-modifying treatment options are still scarce [4]. Pathophysiology of cerebral palsy entails an interplay of multifactorial neuroinflammatory signalling, oxidative stress, excitotoxic neuronal damage, mitochondrial impairment, disrupted synaptic plasticity, and distorted neurodevelopmental pathways [5]. It has been known through molecular studies that cerebral palsy is caused by a disruption of interactive networks of genes and proteins, and is not caused by isolated defects in molecular pathways, which points to the weakness of reductionist therapeutic strategies [6]. Oxidative stress is a cause of neuronal vulnerability that facilitates redox disproportion and cell damage at the crucial times of brain maturation [7]. Continuous inflammatory stimulation only adds to the injury of the nerves and continues to impair the motor capabilities of the affected individuals in the long run [2].

Excitotoxic pathways that are triggered by excessive glutamatergic release cause neuronal death via calcium overload and mitochondrial dysfunction downstream, which results in compromised neural interconnection [8]. A combination of inflammatory cascades and oxidative stress increases neuronal damage and alters the normal developmental pathways in neurons [9]. Traditional pharmacological drugs generally focus on independent biochemical pathways and demonstrate minimal efficacy in a heterogeneous group when treating cerebral palsy phenotypes, which is a divergence between therapeutic design and pathophysiology [3].

Ayurvedic medicine reports several polyherbal preparations that are traditionally used in neurological disorders in children who have motor dysfunction, cognitive impairment, speech delay, and behavioural abnormalities. The Kalyanaka Ghritam is widely mentioned in classic literature regarding the disorders of impaired intellect, language, and neuromuscular control. The formulation incorporates lipid-based processing methods that improve the bioavailability of lipophilic phytoconstituents and enhance absorption, distribution, cellular uptake, and systemic circulation (ADCS) penetration. The experimental data show that phytonutrients in such formulations exhibit antioxidant, anti-inflammatory, neuroprotective, and neuromodulatory properties relevant to the mechanisms of cerebral palsy [10]. Recent applications of network pharmacology in neurological disorders provide supportive evidence for this multi-target paradigm; for instance, studies on Alzheimer's disease and Parkinson's disease have demonstrated that phytochemical compounds can modulate interconnected pathways such as nuclear factor kappa B (NF-κB), mitogen-activated protein kinase (MAPK), and phosphoinositide 3-kinase (PI3K)-Akt signalling, leading to coordinated regulation of neuroinflammation, oxidative stress, and synaptic dysfunction [11,12]. These findings reinforce the relevance of systems-level approaches in complex neurodevelopmental and neurodegenerative conditions.

Network pharmacology has become an integrative model that intertwines systems biology, computational modelling, and pharmacological data to enumerate the multi-component and multi-target activities of complex therapeutic agents [11]. This method will facilitate both the systematic mapping of phytochemical protein interactions and disease-related signalling pathways and the mechanistic assessment of polyherbal preparations [12]. Network-based approaches offer a logical framework for the conversion of conventional medical knowledge into biologically supported hypotheses that can be used in multifactorial neurodevelopmental disorders [13]. For example, network pharmacology-based investigations in Alzheimer's disease have demonstrated that multi-component herbal formulations can simultaneously regulate key signalling pathways, such as PI3K-Akt, MAPK, and NF-κB, leading to coordinated modulation of neuroinflammation, oxidative stress, and synaptic dysfunction, thereby supporting the utility of this approach in complex neurological conditions [12,13].

Although the interest in the use of integrative approaches to neurodevelopmental disorders is increasing, there has been a lack of systematic network pharmacology studies of Ayurvedic preparations in cerebral palsy. Current literature often focuses on individual phytochemicals and neglects to incorporate their molecular targets into the disease-relevant biological networks to allow mechanistic interpretation [11]. The Ayurvedic interventions are mostly associated with symptomatic improvement, reported on clinical observations, which cannot be correlated with the molecular aspects, limiting their acceptance in the context of an evidence-based framework [4].

Kalyanaka Ghritam is an appropriate candidate for network-based evaluation based on its polyherbal formulation, classical neurological signs, and the mentioned neuroactive substances. The possible application of network pharmacology to identify its potential use as an adjunctive therapeutic intervention in cerebral palsy could be to explain its coordinated regulation of neuroinflammatory, oxidative stress, excitotoxic injury, and neurodevelopmental signalling pathways [13]. Kalyanaka Ghritam could be an interesting subject for network-based assessment since it is multi-herbal in composition, has neurological signs of tradition, and has neuroactive phytoconstituents that are reported. The study of its mechanistic relevance via network pharmacology can help to achieve a scientifically consistent explanation of whether it can be used as an adjunctive intervention in the management of cerebral palsy. This synthesis assists in the coordination of the conventional therapeutic frameworks and the state-of-the-art neurobiological theories and responds to the crusades of translation of the modern medical field.

Objectives of the review

This review aims to critically synthesise network pharmacology evidence elucidating the mechanistic relevance of Ayurvedic formulations, with emphasis on Kalyanaka Ghritam, in relation to molecular pathways implicated in cerebral palsy. The analysis seeks to bridge traditional therapeutic concepts with systems-level neurobiological mechanisms to inform translational and clinical perspectives.

Methodology

Literature Search Strategy

The literature search was performed in major databases, including PubMed, Scopus, Web of Science, Google Scholar, and the AYUSH Research Portal, to identify relevant studies published between 2015 and 2025. Search terms were developed using controlled vocabulary and free-text combinations, including cerebral palsy, network pharmacology, systems pharmacology, Ayurvedic formulations, Kalyanaka Ghritam, phytochemicals, neuroinflammation, neuroprotection, and neurodevelopmental pathways. Additional studies were identified through manual screening of the reference lists of eligible articles. Only full-text articles published in English were included to ensure completeness and transparency of data.

Study Selection Process

A structured and transparent approach was adopted for study identification and selection. Records retrieved from databases were screened based on titles and abstracts for relevance, followed by full-text evaluation against predefined inclusion and exclusion criteria. Duplicate records were excluded, and studies not meeting the thematic scope were removed during screening. A total of 41 studies were considered relevant and included in the narrative synthesis based on thematic relevance and mechanistic contribution to the objectives of this review.

Eligibility Criteria

Inclusion criteria: Eligible studies included original experimental studies, in silico network pharmacology analyses, preclinical investigations, and clinical studies examining molecular mechanisms, biological targets, or neurological effects of Ayurvedic herbs or polyherbal formulations relevant to cerebral palsy or related neurodevelopmental disorders. Review articles providing mechanistic insights into network pharmacology in neurological conditions were also considered.

Exclusion criteria: Exclusion criteria comprised studies published prior to 2015, articles lacking mechanistic or molecular relevance, anecdotal reports without analytical rigour, non-peer-reviewed content, conference abstracts, editorials, and studies unrelated to neurological disorders.

Quality Appraisal and Risk of Bias Assessment

Quality appraisal and risk of bias assessment were conducted qualitatively by two independent reviewers, based on study design, methodological rigour, and biological plausibility of reported findings. Discrepancies between reviewers were resolved through discussion to ensure consistency in evaluation. Although a formal quantitative risk-of-bias tool was not applied due to the narrative design, emphasis was placed on peer-reviewed studies with robust experimental or clinical methodology.

Data Extraction and Synthesis

The synthesis of data from eligible studies was conducted using a narrative approach, focusing on phytochemical-target interactions, signalling pathways, and biological networks associated with the pathophysiology of cerebral palsy. Data extraction was guided by predefined thematic domains, including phytochemical composition, molecular targets, signalling pathways, experimental models, and translational relevance, to ensure consistency and reproducibility of interpretation.

## Review

Cerebral palsy: Neurobiological and molecular pathogenesis

Cerebral palsy is a result of developmental problems experienced by the brain in its early stages of development, which leads to impairment of motor control, posture, and coordinated movements [1]. The pathology that underlies is a combination of prenatal and perinatal and early postnatal insults on a susceptible developing nervous system [2]. Hypoxic-ischaemic injury is one of the major initiating factors, especially in infants born preterm and with low birth weight, which causes compromised cerebral blood flow and a lack of oxygen [14]. This injury causes energy deficiency at the cellular level, which leads to neuronal depolarisation, ionic imbalance, and cell death pathways [14]. Selective vulnerability of a cell type of oligodendrocyte precursor cells is a disease mechanism occurring in white matter damage, which is a characteristic of spastic types of cerebral palsy [15]. Neuroinflammation is a focal and persistent pathophysiological event that occurs after the original insult [5]. The stimulation of microglia and astrocytes results in the overproduction of pro-inflammatory cytokines, chemokines, and reactive oxygen species [16]. Higher concentrations of the inflammatory factors include tumour necrosis factor-alpha and interleukin-1 beta, which disrupt the normal neuronal differentiation and maturation of synapses [5]. Constant activation of inflammatory signals during key periods of neurodevelopment alters cortical architecture and motor circuits, thereby strengthening the permanent functional impairment [2]. Excitotoxicity also enhances neuronal damage due to unnecessary discharge of glutamate and an unreasonable activation of ionotropic glutamate receptors [8]. Calcium influx over an extended period of time, triggered by the N-methyl-D-aspartate (NMDA) and α-amino-3-hydroxy-5-methyl-4-isoxazolepropionic acid (AMPA) receptors, induces mitochondrial dysfunction, oxidative stress, and activation of the apoptotic cascade [8]. Both hypoxic injury and excitotoxic signalling have their downstream effects of oxidative stress, which is an accumulation of free radicals and a decrease in antioxidant defence systems [7].

Younger neurons have a poor ability to counter oxidative injury, making brain tissue in development especially vulnerable [17]. Genetic predisposition and epigenetic alteration determine personal susceptibility to cerebral palsy [18]. Alterations in genes that control inflammation, coagulation, mitochondrial processes, and neurodevelopmental signalling have been linked to high risk. Perturbation of essential developmental pathways, including PI3K-Akt, MAPK, and neurotrophin signalling, disrupts neuronal survival, axonal guidance, and synaptic plasticity [18]. These molecular perturbations lead to altered brain connectivity and maladaptive circuit formation to solidify enduring motor and cognitive deficits. Table 1 provides an overview of the main neurobiological processes that are involved in cerebral palsy and related molecular mediators.

**Table 1:** Molecular mechanisms underlying cerebral palsy. HIF-1α: hypoxia-inducible factor 1 alpha, ATP: adenosine triphosphate, Ca²⁺: calcium ion, TNF-α: tumour necrosis factor-alpha, IL-1β: interleukin-1 beta, NF-κB: nuclear factor kappa B, NMDA: N-methyl-D-aspartate, AMPA: α-amino-3-hydroxy-5-methyl-4-isoxazolepropionic acid, ROS: reactive oxygen species, Nrf2: nuclear factor erythroid 2-related factor 2, PI3K: phosphoinositide 3-kinase, MAPK: mitogen-activated protein kinase.

Pathological process	Primary triggers	Key molecular mediators	Cellular consequences	Clinical correlates	References
Hypoxic-ischaemic injury	Reduced oxygen and glucose delivery	HIF-1α, ATP depletion, Ca²⁺ influx	Neuronal death, white matter injury	Spasticity, motor delay (severity correlates with extent of white matter injury on MRI and biomarkers such as lactate accumulation and reduced cerebral oxygenation indices)	[14]
Neuroinflammation	Perinatal infection, tissue injury	TNF-α, IL-1β, NF-κB	Impaired neurodevelopment, gliosis	Cognitive impairment (associated with elevated pro-inflammatory cytokines such as TNF-α and IL-6 in cerebrospinal fluid and serum, correlating with neurodevelopmental outcomes)	[5]
Excitotoxicity	Excess glutamate release	NMDA, AMPA receptors	Mitochondrial dysfunction, apoptosis	Movement disorders (linked with increased glutamate levels and altered neurotransmitter ratios detectable via MR spectroscopy)	[8]
Oxidative stress	ROS overproduction	Nrf2 dysregulation, lipid peroxidation	Cellular damage, synaptic loss	Neurodevelopmental delay (biomarkers include elevated malondialdehyde, decreased superoxide dismutase activity, and impaired antioxidant capacity correlating with functional deficits)	[7]
Genetic susceptibility	Developmental gene variants	PI3K-Akt, MAPK pathways	Altered neuronal signalling	Phenotypic heterogeneity (associated with gene variants affecting neurodevelopmental pathways and epigenetic markers influencing disease severity and clinical variability)	[18]

Rationale for network pharmacology in neurodevelopmental disorders

Stressors in neurodevelopmental disorders involve a complicated and mobile anomaly of multiple molecular pathways, cellular mechanisms, and neural paths in critical periods of maturation of the brain [19]. Other conditions susceptible to neuroinflammation, oxidative balance, synaptic plasticity, mitochondrial functioning, and neurotrophic signalling changes together are cerebral palsy [2]. These mechanisms work with coupled networks of genes and proteins and not single molecular phenomena, signifying dysregulation of neurodevelopmental biology on a systems scale [20]. Traditional pharmacological models based on individual molecular causes do not apply to the distributed character of these pathologies, which can easily end in the restricted efficacy of treatment and unreliable clinical findings [21]. Network pharmacology offers a systems-level paradigm that has the ability to reflect on the complexity of neurodevelopmental disorders [20]. The method combines the pharmacology information with the network of gene-protein interactions, signalling pathways, and molecular signatures associated with diseases [22]. The mapping of interactions among bioactive compounds with various biological targets can be used to identify important network nodes that participate in the modulation of the disease [11]. The analysis can be used to comprehend synergistic and additive effects of multi-component interventions, which are consistent with the biological architecture of emerging neural systems [23].

**Figure 1:**
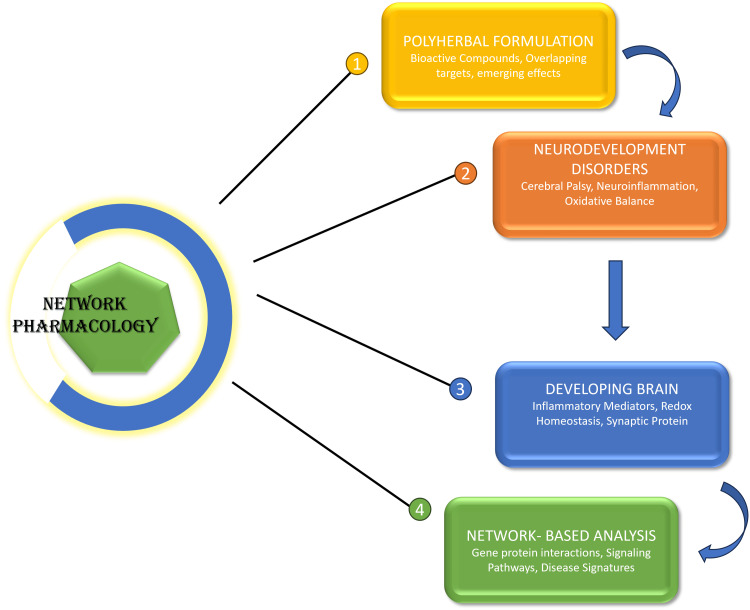
Network-based integration of polyherbal therapeutics in neurodevelopmental pathways. Image credit: Preetham Pai. The figure was created using Microsoft PowerPoint.

The brain is susceptible to varying degrees of simultaneous signalling cascade modulation, which makes the brain more susceptible and plastic to mistakes [2]. Network pharmacology assists in the assessment of how the coordination of inflammatory mediators, redox homeostasis, synaptic proteins, and neurotrophic factors regulates neurodevelopmental outcomes [12]. This integrative approach advocates rational prioritisation of therapeutic measures that have the ability to restore the balance in the network instead of inhibiting individual pathological nodes [19]. Network pharmacology provides a scientifically based approach to the decoding of the intricate phytochemical interactions in the traditional polyherbal formulations [13]. Bioactive compounds in these formulations tend to interact with overlapping targets in many different pathways to produce emergent biological responses that cannot be achieved with monotherapy [24]. The use of network-based analysis helps fulfil a gap between the traditional knowledge of therapy and the latest molecular neuroscience to increase the translational applicability [22]. Implementation of this paradigm promotes the development of multi-target interventions that are appropriate to the multifactorial aetiology of neurodevelopmental disorders [11]. Figure 1 describes network pharmacology as a unification of multi-component herbal treatment with networked brain systems of developing brain molecular pathways.

Ayurvedic polyherbal formulations as multi-component therapeutics

The Ayurvedic polyherbal formulations are based on the notion of a combination of several botanicals with a goal of obtaining a broad spectrum of therapeutic activity due to the coordinated biological action [25]. All the formulations combine herbs with complementary pharmacodynamic effects, which are chosen in order to have an effect on physiological balance, both at the systemic and cellular levels [26]. In contrast to interventions with the specific molecule, polyphenol combinations offer concomitant regulation of a variety of molecular targets, which is an inherently systems-oriented approach to therapy [23]. Such a multi-component structure is specifically applicable to the disorders that have complex and overlapping pathological processes, such as neurodevelopmental and neurological disorders [20]. Synergism is a fallacy in the pharmacological ideology of Ayurvedic preparations [27]. Synergy is an additive, potentiating, or regulatory interaction between phytochemicals found in constituent herbs [24]. Biologically active compounds can increase absorption, stabilise metabolic processes, decrease toxicity, or increase the biological efficacy of other components [25]. Ghrita is also based on lipids that enhance the bioavailability of lipophilic substances and penetrate the central nervous system [26]. These formulation plans allow prolonged and homeostatic adjustment of biological networks as opposed to brief suppression of individual signal transduction pathways [22].

Multifactorial pathogenesis, including neuroinflammation, oxidative stress, excitotoxicity, disrupted synaptic plasticity, and alterations in neurotrophic signalling, is found in central nervous system disorders, especially neurodevelopmental disorders [2]. Relevance polyherbal formulations include simultaneous control of inflammatory mediators, antioxidants of the body, neurotransmitter systems, and neuroprotective pathways [28]. The coordinated activity of a group of nodes of the network helps restore homeostasis of the network, which is consistent with the distributed character of the pathology of the central nervous system [11]. It has been shown through experimentation that several Ayurvedic herbs have pleiotropic effects in signalling cascades, including NF-κB, MAPK, PI3K-Akt, and neurotrophin pathways [10]. These effects, when used in a formulation, interact and result in effects on neuronal survival, glial activation, synaptic remodelling, and mitochondrial functioning [13]. The observed functional improvements in motor coordination, cognition, and behaviour in traditional practice can be explained by such network-level modulation as being mechanistically plausible [21]. A summary of the important properties of Ayurvedic polyherbal preparations in relation to central nervous system conditions is given in Table 2.

**Table 2:** Therapeutic network attributes of Ayurvedic polyherbal formulations. CNS: central nervous system, NF-κB: nuclear factor kappa B, MAPK: mitogen-activated protein kinase, BBB: blood–brain barrier, PI3K: phosphoinositide 3-kinase, BDNF: brain-derived neurotrophic factor, ROS: reactive oxygen species.

Therapeutic principle	Formulation feature	Molecular targets	Biological effects	Relevance to CNS disorders	References
Synergism	Multi-herb composition	NF-κB, MAPK (with contributory phytochemicals such as quercetin, kaempferol, and luteolin known to modulate inflammatory signalling)	Coordinated pathway modulation	Reduced neuroinflammation	[27]
Bioavailability enhancement	Lipid-based carriers	BBB transporters (facilitating delivery of lipophilic terpenoids, sterols, and related neuroactive constituents across neural barriers)	Improved CNS delivery	Enhanced neuroactivity	[26]
Multi-target action	Diverse phytochemicals (including flavonoids, alkaloids, terpenoids, glycosides, and saponins)	PI3K-Akt, BDNF (modulated by compounds such as quercetin, bacoside-like saponins, and other neurotrophic phytoconstituents)	Neuroprotection, plasticity	Motor and cognitive support	[10]
Toxicity mitigation	Balancing herbs	Antioxidant enzymes (supported by phenolic acids, flavonoids, and antioxidant terpenoids that enhance endogenous defence systems)	Cellular homeostasis	Long-term safety	[28]
Network regulation	Pathway convergence	Cytokines, ROS (regulated by polyphenols and alkaloidal constituents acting across inflammatory and redox-sensitive pathways)	Systems-level balance	Multifactorial disease modulation	[11]

Kalyanaka Ghritam: Composition and traditional indications

The Kalyanaka Ghritam is an Ayurvedic lipid-based classical preparation found in the authoritative texts of disorders of the intellect, speech, behaviour, and neuromotor functions [26]. The formulation comprises a complex polyherbal combination traditionally including ingredients such as Haritaki (*Terminalia chebula*), Bibhitaki (*Terminalia bellirica*), Amalaki (*Emblica officinalis*), Haridra (*Curcuma longa*), Daruharidra (*Berberis aristata*), Musta (*Cyperus rotundus*), Vidanga (*Embelia ribes*), Kushtha (*Saussurea lappa*), and other medhya and rasayana botanicals processed in a ghrita (clarified butter) base, although composition may vary slightly across classical texts and formulations [29]. The formulation is made of a blend of botanicals that are processed in clarified butter, which helps in efficient extraction and stabilisation of bioactive phytoconstituents [29]. Ghrita medium increases lipophilic compounds and translocation across neural barriers, a characteristic of special importance to central nervous system modulation [30]. Emerging evidence from Ayurvedic pharmaceutics and drug delivery research suggests that ghrita-based formulations may enhance blood-brain barrier permeability through lipid-mediated transport mechanisms, facilitating improved central nervous system bioavailability of lipophilic phytoconstituents. This property has been proposed as a key factor underlying the neurotherapeutic potential of ghrita preparations in neurodevelopmental and neurodegenerative disorders [26,30]. The herbs used are conventionally chosen as medhya, balya, and rasayana qualities, which are all related to cognitive improvement, neural power, and muscle nutrition [31]. Classical signs of Kalyanaka Ghritam include disorders that are marked by impaired cognition, slow speech acquisition, motor incoordination, as well as disturbed behaviour [26]. The Ayurvedic texts describe the formulation as being used in the treatment of developmental delay, learning disability, susceptibility to seizures, and neuromuscular disability [4]. Such signs are directly related to clinical domains that are impacted in cerebral palsy, such as motor control deficits, speech articulation impairments, deficits in executive functioning, and adaptive behaviour deficits [1]. Its formulation is usually given at the initial stages of the neurological polymerisation, which is consistent with the developmental period when neural plasticity can be altered [32].

Regarding the functional aspects, the botanical Kalyanaka Ghritam has the following pharmacological effects: neuroprotection, anti-inflammatory effects, antioxidant defence, and neuromodulation [10]. The properties assist in stabilisation of neuronal membranes, regulation of neurotransmitter balance, and inhibition of inflammatory mechanisms that disrupt normal brain maturation [7]. The holistic composition allows simultaneous involvement of the various physiological systems, which is one of the approaches to the disorders with multifactorial pathogenesis [20]. The conceptual congruence between the classical description of symptoms and modern neurodevelopmental constructs becomes relevant in concepts of cerebral palsy [3]. The classical sources describe motor stiffness, speech delay, impaired cognition, and behavioural dysregulation in people with cerebral palsy, which are similar to those of contemporary clinical descriptions of the condition [1]. Combined with the conventional reflections and molecular and network-based interpretations, the integration provides a consistent scheme of assessing Kalyanaka Ghritam in the framework of contemporary neurotherapeutic models [22].

Phytochemical constituents of Kalyanaka Ghritam

Kalyanaka Ghritam constitutes a wide range of phytochemicals that are the products of its constituent botanicals and make it have wide neurobiological activity [29]. Analytical research on the individual herbs used in the formulation has discovered alkaloids, flavonoids, terpenoids, saponins, phenolic acids, and glycosides as the major bioactive classes [28]. The compounds have a high pharmacological versatility and are involved in the modulation of various molecular targets that are involved in the functioning of the central nervous system [33]. Lipid medium processing improves the extraction activity and stability of lipophilic phytoconstituents, which makes them available to prolonged biological activity after administration [30]. The antioxidant and anti-inflammatory effects of flavonoids, such as quercetin, kaempferol, and luteolin, have been reported in several constituent herbs [28]. These compounds control redox homeostasis by neutralising reactive oxygen species and stimulating the endogenous antioxidant mechanisms [34]. Simultaneous inhibition of pro-inflammatory signalling pathways helps in the inhibition of microglial activation and cytokine release, which are involved in neurodevelopmental injury [35]. The presence of terpenoids and steroidal compounds in the formulation has been reported to have a membrane-stabilising and mitochondrial protective effect that facilitates neuronal energy metabolism and cellular resilience [17].

The presence of alkaloids and glycosidic compounds in the identified ingredients has neuromodulatory effects caused by the interaction of the products with neurotransmitter systems and ion channels [8]. The cholinergic, glutamatergic, and GABAergic signalling are regulated to aid in the maintenance of synaptic balance and neuronal excitability [9]. This modulation is also relevant to motor coordination and cognitive processing, which are often impaired in neurodevelopmental disorders [4]. Saponins and phenolic acids also play a role in neuroprotective activity by improving neurotrophic factor expression and preventing apoptotic mechanisms [36]. The phytochemical diversity of Kalyanaka Ghritam allows the antioxidant defence mechanisms, inflammatory control, synaptic regulation, and neurotrophic support to be achieved simultaneously [10]. This type of multi-dimensional activity profile corresponds to the therapeutic needs of complex neurological conditions at the system level [20]. The combination of these phytochemicals into one formulation facilitates network modulation as opposed to isolated molecular intervention, which offers a mechanistic plausibility of conventional neurological indication [22]. Table 3 shows representative phytochemical-target interactions underlying the neuroprotective and neuromodulatory mechanisms of Kalyanaka Ghritam.

**Table 3:** Representative phytochemical-target interactions underlying neuroprotective mechanisms of Kalyanaka Ghritam. NF-κB: nuclear factor kappa B, Nrf2: nuclear factor erythroid 2-related factor 2, MAPK: mitogen-activated protein kinase, PI3K: phosphoinositide 3-kinase, BDNF: brain-derived neurotrophic factor, AMPA: α-amino-3-hydroxy-5-methyl-4-isoxazolepropionic acid, NMDA: N-methyl-D-aspartate, TNF-α: tumour necrosis factor-alpha, IL-1β: interleukin-1 beta, ROS: reactive oxygen species, SOD: superoxide dismutase.

Phytochemical	Class	Molecular targets	Mechanistic action	Relevance to cerebral palsy	References
Quercetin	Flavonoid	NF-κB, Nrf2	Anti-inflammatory, antioxidant activation	Reduces neuroinflammation and oxidative stress	[28,34]
Kaempferol	Flavonoid	MAPK, PI3K-Akt	Modulates cell survival and inflammation	Supports neuronal survival and plasticity	[28]
Bacoside-like saponins	Saponin	BDNF, synaptic proteins	Enhances neurotrophic signalling	Promotes neurogenesis and synaptic plasticity	[36]
Berberine-like alkaloids	Alkaloid	AMPA/NMDA receptors, cholinergic pathways	Neuromodulation, excitotoxicity reduction	Improves neurotransmission balance	[8,9]
Terpenoids	Terpenoid	Mitochondrial enzymes, ROS pathways	Membrane stabilisation, antioxidant effect	Protects mitochondrial function	[17]
Phenolic acids	Polyphenol	Antioxidant enzymes (SOD, catalase)	ROS scavenging	Supports redox homeostasis	[34]

**Figure 2:**
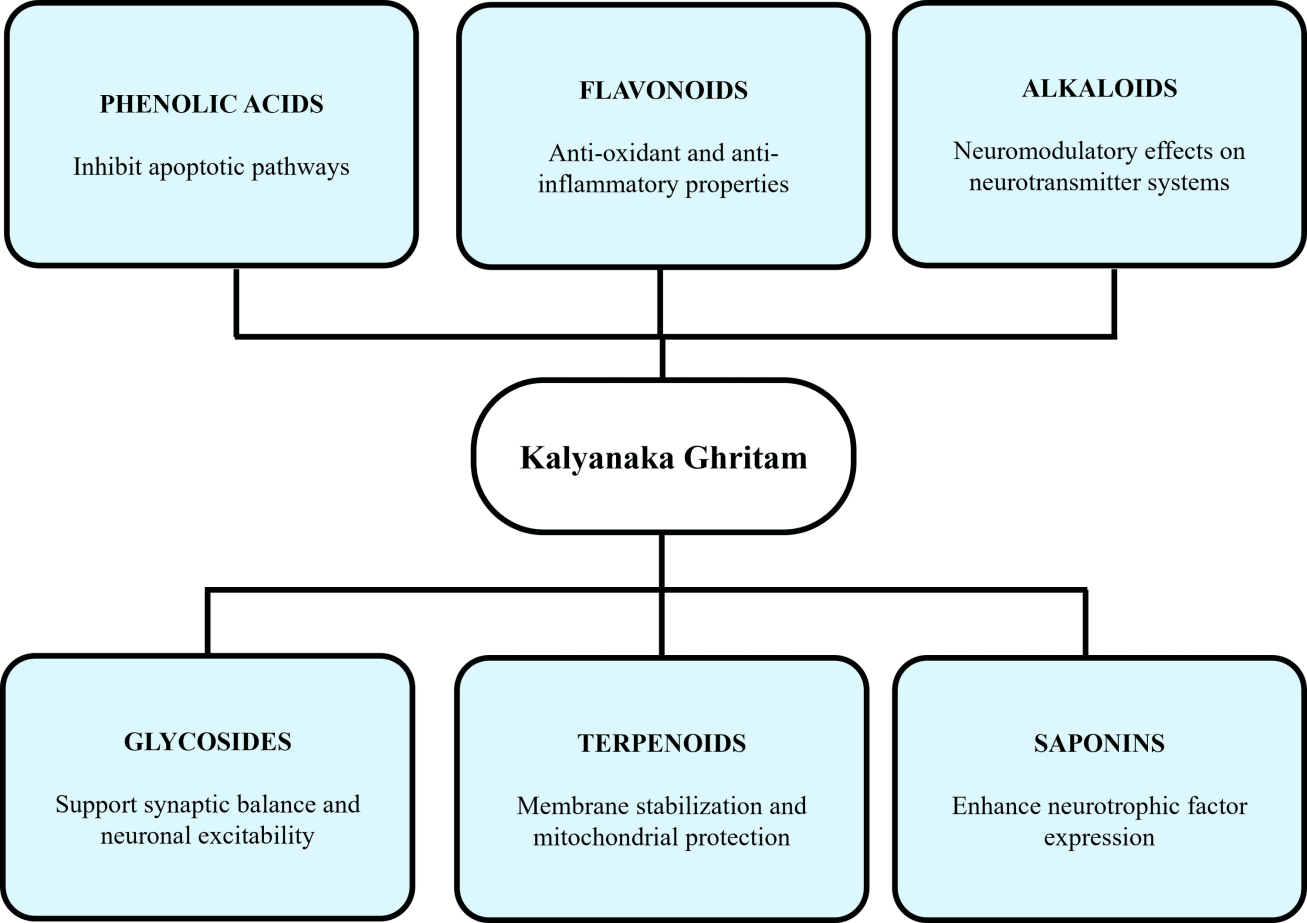
Phytochemical-driven neurobiological actions of Kalyanaka Ghritam. Image credit: Preetham Pai. The figure was created using Microsoft PowerPoint.

Figure 2 indicates the major phytochemical classes in Kalyanaka Ghritam and the synthesised neuroprotective and neuromodulatory effects.

Target gene and protein network interactions

Interactions between target genes and protein networks give a mechanistic basis for comprehending the impacts of multi-component formulations on complex neurological diseases [20]. The bioactive phytochemicals identified in Kalyanaka Ghritam by network pharmacology imply that several genes and proteins involved in the pathophysiology of cerebral palsy interact with them [12]. These are targets of inflammatory mediators, redox regulators, neurotrophic factors, and intracellular signalling molecules that are interconnected biological networks, and not separate linear pathways [22]. Necessary inflammatory controllers, such as NF-κB-related signalling proteins, are important nodes in these interaction networks [37]. NF-κB modulation controls the expression of pro-inflammatory cytokines and adhesion molecules, which help to regulate neuroinflammatory tone at critical times in the development of the brain [5]. Simultaneous activation of antioxidant response components that Nrf2 controls helps the cell to respond to oxidative stress by activating the expression of endogenous detoxifying enzymes [34]. Integrated control over inflammatory and redox processes is beneficial to neuronal survival and white matter preservation [38].

Another key element of the interaction network, as predicted, is neurotrophic signalling [10]. BDNF-mediated pathways are proteins that affect neuronal differentiation, synaptic plasticity, and circuit maturation [32]. Phytochemicals-BDNF-related signal interaction facilitates activity-dependent remodelling of synapses and linkage in motor and cognitive networks [36]. These effects are pertinent to neurological outcomes of cerebral palsy over an extended period of time [1]. Extracellular signals are combined with transcriptional and metabolic reactions through intracellular signalling cascades that comprise MAPK and PI3K-Akt pathways [19]. The interactions that occur between the networks about these pathways control cell survival, mitochondrial activity, and the development of axons [14]. Concomitant regulation of MAPK and PI3K-Akt signalling facilitates the balanced regulation of apoptosis and neuroprotection, which are in line with systems-level therapeutic goals [8]. These pathways also overlap with the inflammatory and neurotrophic pathways, supporting the biological coordination [9].

**Figure 3:**
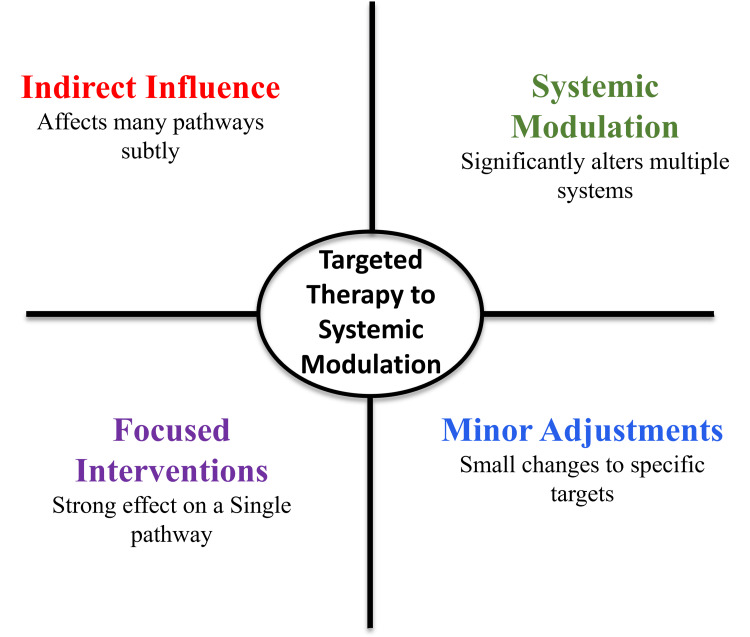
Shift from targeted intervention to systemic network modulation. Image credit: Preetham Pai. The figure was created using Microsoft PowerPoint.

All these interactions between target genes and protein networks related to Kalyanaka Ghritam point to the existence of a multi-node regulatory structure with the potential to control a wide range of pathological mechanisms that play a role in causing cerebral palsy [18]. This kind of network-level interaction facilitates the mechanistic plausibility of multi-target intervention strategies to neurodevelopmental disorders [13]. Figure 3 shows a transition from focused single-target intervention to indirect systemic modulation within interconnected biological networks.

Modulation of neuroinflammation and oxidative stress pathways

Neuroinflammation and oxidative stress are mutually reinforcing pathological mechanisms that play a major role in the genesis and maintenance of a state of neurological dysfunction in cerebral palsy [5]. Brain injury at an early stage induces long-term elevation of microglia and astrocyte activation, which results in the overproduction of pro-inflammatory cytokines, chemokines, and reactive nitrogen and oxygen species [35]. The ongoing inflammatory state disrupts both oligodendrocyte differentiation, axonal myelination, and synaptic structure, contributing to chronic motor and cognitive dysfunction [15]. Unregulated redox homeostasis also enhances tissue injury by increasing lipid peroxidation, protein oxidation, and mitochondrial injury [17]. Kalyanaka Ghritam is linked with phytochemicals that have the ability to modulate both the inflammatory and oxidative processes by depending on multi-target interactions [10]. Analyses based on networks suggest that network-based mediation of cytokine signalling cascades of tumour necrosis factor-alpha and interleukin-1 beta, the major mediators of neuroinflammation by microglia, is modulated [37]. These mediators are attenuated, and this leads to loss of glial reactivity and preserves the integrity of the neuronal microenvironment [39]. The Cdk5 of transcription factors like NF-κB also controls transcription factor propagation of inflammatory genes, which sustains regulated immune signalling into the developing brain [6].

Endogenous antioxidant systems are involved in oxidative stress control by operating simultaneously [34]. Nrf2-regulated pathways are triggered, which boosts the expression of enzymes that eliminate reactive oxygen species, such as superoxide dismutase and catalase [7]. Antioxidant capacity recovery preserves neuronal membranes and mitochondrial activity, reducing the effects of energy malfunction and apoptotic signalling [36]. Redox stabilisation of this type promotes the survival of neurons and their synapses; these processes are critical to the integrity of motor circuits [14]. The interaction of inflammatory and oxidative processes is consistent with the multifactorial pathology of spasticity and developmental delay in neurodevelopment [3]. Cerebral palsy clinical manifestation is related mechanistically to chronic inflammation and oxidative damage to the integrity of the corticospinal tract and neuromuscular signalling [1]. Neuroinflammatory decrease helps to maintain good motor neuron conduction and muscle tone control [40]. Oxidative damage suppression at the network level also helps in conservation of neural plasticity under developmental stages when neural functions are most crucial towards functional recovery [32]. Table 4 summarises vital inflammatory and oxidative pathways regulated by multiple components relevant to cerebral palsy.

**Table 4:** Network modulation of neuroinflammation and oxidative stress in cerebral palsy. TNF-α: tumour necrosis factor-alpha, IL-1β: interleukin-1 beta, NF-κB: nuclear factor kappa B, Nrf2: nuclear factor erythroid 2-related factor 2, ROS: reactive oxygen species.

Pathway category	Key molecular targets	Modulatory action	Cellular outcome	Clinical relevance	References
Neuroinflammation	TNF-α, IL-1β	Cytokine suppression	Reduced glial activation	Improved motor control	[37]
Transcriptional regulation	NF-κB	Inhibition of inflammatory gene expression	Neuroimmune balance	Reduced spasticity	[6]
Oxidative stress response	Nrf2	Antioxidant enzyme activation	Redox stabilization	Neuroprotection	[34]
Mitochondrial integrity	ROS regulators	Free radical scavenging	Energy preservation	Developmental support	[7]
Neuro-glial interaction	Microglial mediators	Immune modulation	Synaptic stability	Functional improvement	[35]

Effects on synaptic plasticity, neurogenesis, and neuroprotection

Synaptic plasticity, neurogenesis, and neuroprotection form the basis of processes involved in functional recovery and adaptive capacity in neurodevelopmental disorders [32]. In cerebral palsy, brain impairment that occurs in the early stages interferes with the refinement of synapses, changes the equilibrium of neurotransmitters, and impairs the survival of neurons in motor and associating cortices [2]. These changes disrupt the processes of neural circuit formation and stabilisation that instruct the movement, cognition, and learning [1]. Recovery of cellular and synaptic integrity is also necessary in the long-term neurological recovery [3]. The mechanistic relevance of bioactive compounds related to Kalyanaka Ghritam in synaptic modulation has been shown with respect to the regulation of neurotransmission and expression of synaptic proteins [10]. The excitatory-inhibitory balance in the developing neural networks is facilitated by the modulation of glutamatergic and GABAergic signalling [8]. Synaptic receptors and ion channels stabilisation help in enhancing the transmission of signals and inhibiting maladaptive firing patterns of neurons [9]. The effects of this sort are more synaptic remodelling processes necessary in motor coordination and cognitive integration [4]. Recent experimental and animal model studies further support these mechanisms, demonstrating that phytochemical-rich interventions targeting PI3K-Akt, MAPK, and neurotrophin pathways enhance synaptic protein expression, dendritic spine density, and functional recovery following neonatal brain injury and hypoxic-ischaemic models relevant to cerebral palsy [8,14]. Such studies also report improved motor coordination, reduced neuroinflammation, and restoration of neurotransmitter balance, reinforcing the translational relevance of multi-target phytochemical modulation.

Signalling pathways that affect neurogenesis and neuronal differentiation include progenitor cell proliferation and maturation [14]. Phytochemicals that are connected with the formulation interrelate with the neurotrophic pathways that relate to the growth of neurons and neuronal survival [36]. Improvement of neurotrophin-mediated signalling assists dendritic arborisation, axonal growth, and synaptic affiliation [10]. These mechanisms lead to structural and functional rearrangement of the damaged neural circuits that promote compensatory plasticity during development [32]. Some of these neuroprotective mechanisms include mitochondrial preservation, modulation of apoptotic signalling, and cellular homeostasis [17]. Antioxidant and anti-inflammatory effects linked to the constituent compounds lower the cellular stress and deter the progressive neuronal loss [7]. Preservation of oligodendrocytes and neurons assists in the preservation of the integrity of myelinated tracts and synaptic transmission [15]. This type of cellular preservation is the basis of long-term neuromotor and cognitive gains [1]. The interaction of synaptic plasticity, neurogenesis, and neuroprotection constitutes a biological context where adaptive neural remodelling can be established [19]. Coordinated adjustment of such processes is consistent with the multifactorial treatment needs of cerebral palsy, which offers a mechanistic explanation of multi-target interventions with the capacity to affect developmental pathways [13].

Translational relevance and clinical evidence

Such relevance of multi-component formulations to the translational applicability in cerebral palsy requires conformity of mechanistic plausibility, preclinical validation, and clinical outcomes of the study [13]. Neuroprotective, anti-inflammatory, antioxidant, and neuromodulatory effects on cellular and animal models of neurological injury are demonstrated by experimental studies of individual botanical constituents of Kalyanaka Ghritam [36]. According to such studies, attenuation of neuronal apoptosis, mitochondrial functional preservation, glial activation attenuation, and motor coordination and cognitive performance improvements are reported [39]. The results are consistent with biological processes that apply to hypoxic-ischaemic damage, neuroinflammation, and disrupted synaptic maturation related to cerebral palsy [14]. Evidence supporting the use of multi-target modulation in neonatal brain injury-related animal models shows that this approach improves neuronal survival and functional recovery [41]. There has been an increase in locomotor, muscle tone regulation, and learning behaviour associated with the administration of neuroactive phytochemicals acting on inflammatory and oxidative pathways [39]. Recruitment of signalling cascade pathways, including PI3K-Akt, MAPK, and neurotrophin in neuroplastic responses at network levels, contributes to adaptive remodelling of neural circuitry during development stages [8]. The results of this kind of work reinforce the plausibility of the translation of formulations that seek to modify complex neurodevelopmental networks [20].

Kalyanaka Ghritam has limited clinical evidence in relation to it, as it is mostly observational [31]. Conventional clinical practice shows functional improvements in speech, cognition, motor coordination, and behavioural control after extended usage in paediatric neurological disorders [26]. However, there is a notable lack of randomised controlled trials (RCTs) and large-scale clinical datasets specifically evaluating Kalyanaka Ghritam or comparable Ayurvedic formulations in cerebral palsy, which limits the strength of clinical inference and evidence-based acceptance. Existing evidence is largely derived from observational studies, preclinical models, and extrapolation from related neurodevelopmental conditions, underscoring the need for rigorously designed controlled trials with standardised outcome measures. Ameliorations are often explained together with such forms of supportive treatment as physiotherapy and speech intervention, indicating an adjunctive therapeutic role [4]. There is a lack of standardised outcome measures and controlled study designs to make a conclusive attribution of efficacy [21]. Traditional usage suggests good tolerability with safety regarding its use under monitored conditions and appropriate dosage depending on age [29]. The lipid-based formulation promotes a slow pharmacological effect, and low chances of acute adverse effects are also reduced [30]. Formulation composition, dosage guidelines, and duration of treatment should continue to be standardised to be incorporated into evidence-based systems [24]. To determine therapeutic relevance in the contemporary paradigms of therapy of cerebral palsy, there is a need to consolidate the preclinical mechanistic data with rigorously designed clinical research [1].

Safety, standardisation, and integration into contemporary care

The crucial factors in the integration of traditional formulations into modern palliative management of cerebral palsy include safety and quality assurance [1]. Polyherbal preparations are characterised by a large number of bioactive compounds that are able to affect various physiological systems, and toxicological evaluation must be carefully carried out [25]. Available experimental evidence on constituent herbs of Kalyanaka Ghritam has shown that it is not very toxic with therapeutically relevant doses, with a long history of clinical use in paediatric populations [31]. Preclinical toxicological assessments of constituent herbs and ghrita-based formulations generally indicate a high safety margin, with no significant organ toxicity observed at standard therapeutic doses; traditionally recommended paediatric dosing ranges are typically in the order of 2.5-10 mL/day (adjusted according to age, weight, and clinical condition), administered under medical supervision, although precise dose standardisation remains variable across studies and classical references [26,29]. No cases of acute toxicity and few cases of adverse events reported in observational studies indicate that there is an acceptable safety margin when administering under supervision [26]. Constant observation is also significant in the neurological treatment of children since the treatment period may be long [4]. The standardisation of lipid-based polyherbal formulations is a very grave problem [24]. The differences in raw botanical sources, methods of processing, and phytochemical levels affect pharmacological reproducibility [23]. Reproducibility and quality control are facilitated by the adoption of good manufacturing practices, botanical authentication, and phytochemical fingerprinting [22]. Determination of marker compounds and evaluation of lots-to-lots consistency increase reliability and enable regulatory acceptance [29]. Ghrita-based preparations, also subjected to stability testing, help in the preservation of bioactive components during storage and administration [30].

To become part of modern cerebral palsy management, it is necessary to fit it into the multidisciplinary care model that focuses on rehabilitation, pharmacological support, and education of caregivers [3]. Kalyanaka Ghritam has a prospective value as an adjunctive intervention to physiotherapy, occupational therapy, speech therapy, and nutritional management [21]. Multi-target biological activity aligns with the objectives of holistic care, which aim at motor functioning, cognition, and behavioural control [10]. Adjunct application does not substitute known evidence-based interventions but promotes personalised interventions [1]. Standardised dosing, age-specific formulations, and treatment endpoints also play a part in clinical integration [19]. Integration of traditional medicine practitioners and biomedical clinicians helps in delivering care and patient safety in a coordinated manner [33]. It is observed that functional engagement and quality of life would improve when there is an integration of integrative approaches in hierarchical clinical approaches [26]. The future progress of wider clinical adoption relies on the creation of high-quality evidence, regulatory alignment, and further focus on patient-centred safety and efficacy testing [13].

Limitations and future recommendations

The comprehension of the results is limited to the dependence on non-homogeneous preclinical, in silico, and observational sources instead of controlled clinical studies of cerebral palsy. Predictions of network pharmacology rely on the completeness of the database and assumptions of algorithms, which restrain biological validations directly. The formulation composition, phytochemical concentrations, and traditional preparation processes vary, which limits reproducibility and comparability across studies. The lack of standardised outcome measures and age-specific clinical information further limits the transfer into the therapeutic guidance provided on an evidence-based basis.

The future research must focus more on well-planned experimental research to confirm the predicted molecular targets and pathway interactions in relation to cerebral palsy. The use of longitudinal clinical studies, normal formulations, dose schedules, and objective neurodevelopmental outcomes is needed. A combination of the omics-based strategies with the network pharmacology can fine-tune the mechanistic resolution. Regulatory frameworks that foster quality control and interdisciplinary clinical collaboration of multi-component Ayurvedic interventions to boost the translational potential and clinical integration will be developed.

## Conclusions

Cerebral palsy is a complicated neurodevelopmental disorder that is instigated by a combination of molecular, cellular, and network-level abnormalities that cannot be reduced to single-target treatment methods. Network pharmacology provides a scientifically adequate paradigm of understanding multi-component Ayurvedic preparations in current appreciations of neurobiological models. The outcome of the synthesis of the microarray suggests that Kalyanaka Ghritam activates several biological pathways involved in cerebral palsy, such as neuroinflammatory regulation, oxidative stress regulation, synaptic plasticity, neurogenesis, and neuroprotection. The phytochemical variety of application and lipid-based formulations properties endorse the orchestrated gathering of the networks of interactions between genes and proteins associated with neurodevelopmental well-being. This kind of systems-level involvement is consistent with the multifactorial aetiology of cerebral palsy and facilitates its inclusion as an integrative care adjunctive intervention. Converging computational predictions, preclinical observations, and conventional clinical use provide support of translational relevance, yet gaps in evidence exist. Standardisation, safety testing, and substantive clinical validation are critical preconditions of greater acceptance. Network pharmacology integration with pre-existing therapeutic expertise helps fill the gap between empirical practice and contemporary systems biology to promote the rational investigation of multi-target interventions to fit complex neurodevelopmental disorders.
